# Lentivirus-Mediated BCL-X_L_ Overexpression Inhibits Stem Cell Apoptosis during Ex Vivo Expansion and Provides Competitive Advantage Following Xenotransplantation

**DOI:** 10.3390/ijms25074105

**Published:** 2024-04-07

**Authors:** Patricia M. A. Zehnle, Ying Wu, Naile Koleci, Sheila Bohler, Miriam Erlacher

**Affiliations:** 1 Division of Pediatric Hematology and Oncology, University Medical Center Freiburg, 79106 Freiburg, Germany; 2 Division of General Pediatrics, University Medical Center Freiburg, 79106 Freiburg, Germany

**Keywords:** hematopoietic stem cells, apoptosis, BCL-2, BCL-X_L_, caspases

## Abstract

Hematopoietic reconstitution after hematopoietic stem cell transplantation (HSCT) is influenced by the number of transplanted cells. However, under certain conditions donor cell counts are limited and impair clinical outcome. Hematopoietic stem and progenitor cell (HSPC) expansion prior to HSCT is a widely used method to achieve higher donor cell counts and minimize transplantation-related risks such as graft failure or delayed engraftment. Still, expansion in a non-physiological environment can trigger cell death mechanisms and hence counteract the desired effect. We have shown earlier that during HSCT a relevant amount of HSPCs were lost due to apoptosis and that cell death inhibition in donor HSPCs improved engraftment in xenotransplantation experiments. Here, we assessed the effect of combined ex vivo expansion and cell death inhibition on HSPC yield and their reconstitution potential in vivo. During expansion with cytokines and the small molecule inhibitor StemRegenin 1, concomitant lentiviral overexpression of antiapoptotic BCL-X_L_ resulted in an increased yield of transduced HSPCs. Importantly, BCL-X_L_ overexpression enhanced the reconstitution potential of HSPCs in xenotransplantation experiments in vivo. In contrast, treatment with caspase and necroptosis inhibitors had no favorable effects on HSPC yields nor on cell viability. We postulate that overexpression of antiapoptotic BCL-X_L_, both during ex vivo expansion and transplantation, is a promising approach to improve the outcome of HSCT in situations with limited donor cell numbers. However, such apoptosis inhibition needs to be transient to avoid long-term sequelae like leukemia.

## 1. Introduction

Hematopoietic stem cell transplantation (HSCT) to date remains the only curative treatment option for a multitude of hematological and immunological disorders but is accompanied by transplantation-related morbidity and mortality. During the period of pancytopenia, infections and bleeding represent severe complications. To assure timely blood cell recovery, a sufficient number of available donor hematopoietic stem and progenitor cells (HSPCs) is substantial. Under certain conditions, however, donor cell counts are limited, for example if umbilical cord blood transplants are the only donor option for adult patients or when there are significant weight differences between pediatric patients and their matched siblings. These situations call for strategies to increase the numbers of transplantable HSPCs and ensure successful hematopoietic regeneration. Different combinations of hematopoietic cytokines, usually comprising FMS-like tyrosine kinase 3 ligand (FLT3L), thrombopoietin (TPO), and stem cell factor (SCF), have been widely used for in vitro expansion of HSPCs prior to HSCT [1,2]. Other approaches rely on small molecules, such as the aryl hydrocarbon receptor antagonist StemRegenin 1 (SR-1). SR-1 was shown to enhance the expansion of human hematopoietic stem cells (HSCs) while preserving their multi-lineage potential [3]. The compound had been tested in a clinical phase I trial with promising results [4].

Apoptosis inhibition in donor HSPCs represents a novel concept to increase the amount of HSPCs contributing to hematopoietic regeneration. This concept is based on the hypothesis that during the entire transplantation procedure HSPCs lack prosurvival signals normally provided by their physiological microenvironment, the stem cell niche [5]. Indeed, we have shown earlier that a relevant amount of HSPCs die during HSCT and that this cell death is mediated via the proapoptotic BCL-2 proteins BIM and BMF [6]. Furthermore, knockdown of either BIM or BMF in human CD34^+^ cells enhanced hematopoietic engraftment in a competitive reconstitution setting in vivo. A similar effect was achieved by overexpression of their antagonists BCL-2 or BCL-X_L_ [6]. To avoid long-term side effects of apoptosis inhibition, we developed a method to transiently inhibit apoptosis during HSCT by delivery of BCL-X_L_ via a cell-penetrating peptide. While short-term apoptosis inhibition was sufficient to improve HSCT outcome in the murine system, no efficient protein transduction could be achieved in human CD34^+^ cells [7].

Here we hypothesize that the combination of different strategies might result in optimal stem cell yields with cell death inhibition improving cell survival during both ex vivo expansion and engraftment. Indeed, we demonstrate that lentiviral overexpression of antiapoptotic BCL-X_L_ is able to enhance numbers of human CD34^+^ cells expanded in vitro. However, both SR-1-mediated expansion and lentiviral BCL-X_L_ overexpression did not measure up to our expectations. SR-1 only resulted in mild expansion of CD34^+^ cells and the use of lentiviruses was accompanied by strong toxicity. Caspase and necroptosis inhibitors, which were used alternatively, exerted no beneficial effects during expansion. In xenotransplantation experiments in vivo, however, BCL-X_L_-overexpressing, expanded HSPCs showed an increased reconstitution potential. We therefore consider the combined approach useful, but other strategies, both for ex vivo expansion and for apoptosis inhibition, with more efficiency and less toxicity should be applied. Importantly, apoptosis inhibition needs to be restricted to the time frame of in vitro expansion and early engraftment to prevent malignant transformation.

## 2. Results

### 2.1. Apoptosis of CD34^+^ Cells Occurs during Ex Vivo Expansion

**Figure 1:**
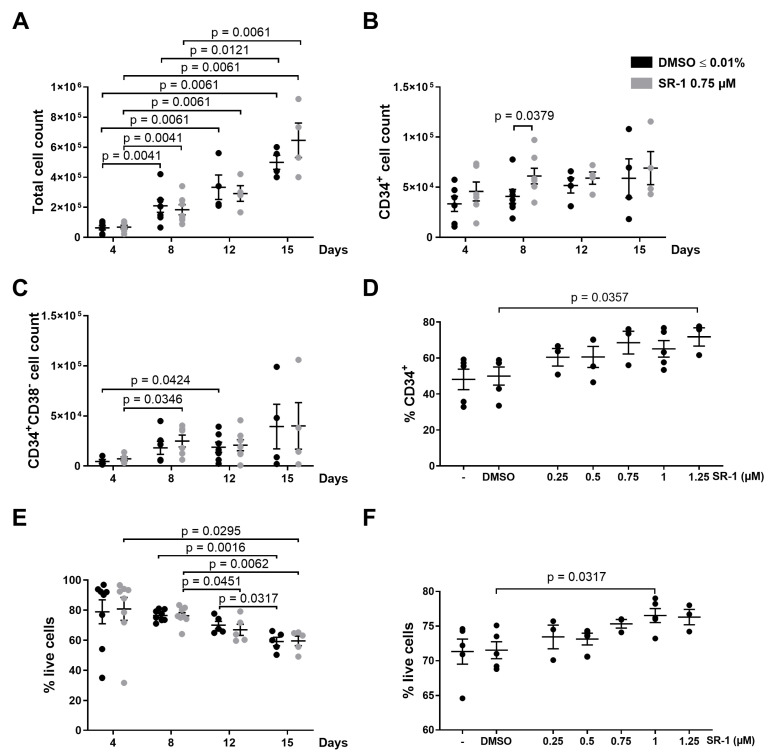
Expansion of CD34^+^ cells by cytokines and StemRegenin 1. 10^4^ CD34^+^ cells were cultured in the presence of cytokines (100 ng/mL thrombopoietin (TPO), stem cell factor (SCF), FMS-like tyrosine kinase 3 ligand (FLT3L), and interleukin-6 (IL-6)) and treated with StemRegenin 1 (SR-1) at the indicated concentrations or DMSO 0.01% (control). Cells were counted and analyzed by flow cytometry at the indicated time points. The amount of live cells was determined by staining with 7-AAD and Annexin V. (**A**): Total cell count. (**B**): CD34^+^ cell count. (**C**): CD34^+^CD38^−^ cell count. (**D**): % CD34^+^ was determined on day 4 of culture. (**E**): % of live cells. (**F**): % of live cells on day 8 of culture. Bars represent means of *n* = 4–7 from 6 independent experiments ± SEM for (**A**–**C**), means of *n* = 3–5 from 4 independent experiments ± SEM for (**D**,**F**), and means of *n* = 5–8 from 7 independent experiments ± SEM for (**E**). Significant *p* values are indicated (Mann–Whitney Test).

In order to expand HSPCs, we followed a protocol published by Boitano et al. [3]. Briefly, HSPCs were expanded by the aryl hydrocarbon receptor antagonist SR-1 in the presence of the cytokines TPO, SCF, FLT3-L, and interleukin-6 (IL-6). Even though the presence of SR-1 in the culture medium did not affect the total cell count (Figure 1A), it specifically had an influence on the CD34^+^ cell count, with significantly increased CD34^+^ cell counts on day 8 (Figure 1B). The immature CD34^+^CD38^−^ cell population was not affected by SR-1 treatment (Figure 1C). With the aim to optimize the expansion protocol, different SR-1 concentrations were used but did not result in a stronger expansion of CD34^+^ cells (68.5% CD34^+^ cells in 0.75 µM SR-1 vs. 71.7% in 1.25 µM; Figure 1D). In addition, different media were tested based on the protocol published by Boitano et al. [3] but also did not enhance the effects of the compound.

The viability of cultured cells declined over time, with a viability of around 60% after 2 weeks of culture, independently of the presence or absence of SR-1 (Figure 1E). Higher SR-1 concentrations resulted in a somewhat increased cell viability (71.5% live cells in DMSO control vs. 76.5% in SR-1 1 µM, *p* = 0.0317; Figure 1F).

### 2.2. CD34^+^ Cells Overexpressing BCL-X_L_ Accumulate during Ex Vivo Expansion

**Figure 2:**
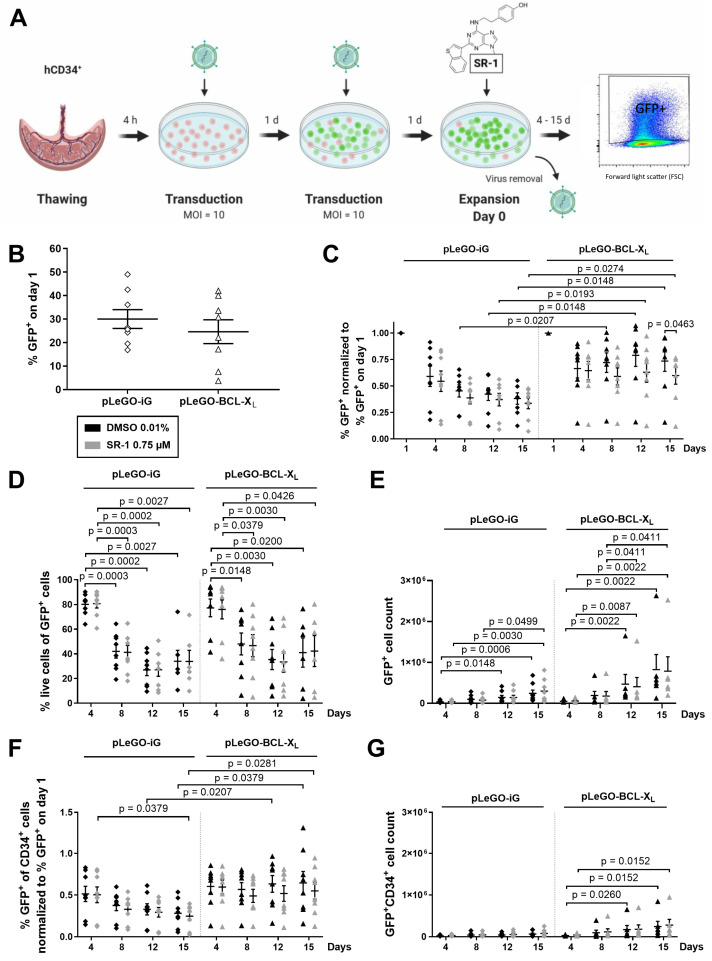
BCL-X_L_ overexpressing CD34^+^ cells accumulate in vitro. (**A**) Experimental design of the combination of ex vivo stem cell expansion culture with lentiviral BCL-X_L_ overexpression. 10^4^ CD34^+^ cells were transduced twice with pLeGO-iG (control), pLeGO-BCL-X_L_ lentivirus (MOI = 10), or remained untreated. Subsequently, the cells were cultured in the presence of cytokines (100 ng/mL TPO, SCF, FLT3L, and IL-6) and treated with 0.75 µM SR-1 or DMSO 0.01% (control). Cells were counted and analyzed by flow cytometry at the indicated time points. Experiments with <10% GFP^+^ on day 1 were excluded from the analyses of absolute cell counts (**E**,**G**). (**B**) The transduction efficiency was determined by flow cytometry on day 1. (**C**) % GFP^+^ at the indicated time points was normalized to % GFP^+^ on day 1. (**D**) % of live cells of the GFP^+^ population was determined by staining with 7-AAD and Annexin V. (**E**) GFP^+^ cell count. (**F**) % GFP^+^ of CD34^+^ cells at the indicated time points was normalized to % GFP^+^ on day 1. (**G**) GFP^+^ CD34^+^ cell count. Bars represent means of *n* = 8 from 8 independent experiments ± SEM for (**B**,**C**,**F**), means of *n* = 6–8 from 8 independent experiments ± SEM for (**D**), and means of *n* = 8 for pLeGO-iG and *n* = 6 for pLeGO-BCL-X_L_ from 8 independent experiments ± SEM for (**E**,**G**). Significant *p* values are indicated (Mann–Whitney Test).

To test whether the HSPC expansion can be improved by concomitant apoptosis inhibition, we stably overexpressed the antiapoptotic BCL-2 protein BCL-X_L_ in human CD34^+^ cells. GFP expression was used to track the transduced cells. After lentiviral transduction (2 × 24 h, MOI 10 each), cells were cultured as described above (Figure 2A). The median transduction efficiency of pLeGO-iG, a control virus expressing only GFP, and pLeGO-BCL-X_L_ virus in CD34^+^ cells was comparable with 30.0% and 24.6%, respectively (Figure 2B). Because of the high variability of the transduction efficiency, we normalized the % GFP expression for our later analyses (Figure 2C,F). Furthermore, two experiments with a transduction efficiency of <10% were excluded when absolute cell counts were determined (Figure 2E,G).

Figure 2C shows a gradual decline of GFP^+^ cells when they were transduced with the control virus, pLeGO-iG, indicating that lentiviral transduction resulted in a reduced fitness. In contrast, the fraction of BCL-X_L_ overexpressing GFP^+^ cells did not drop but remained at a significantly higher level. Surprisingly, the viability of the transduced cells decreased over time in both groups (Figure 2D). Treatment with SR-1 did not affect the percentage of GFP^+^ cells nor their viability (Figure 2C,D). In sum, the yield of total cells was mildly, but not significantly, increased when BCL-X_L_ was overexpressed (Figure 2E). Analysis of the immature CD34^+^ cells revealed a similar loss of GFP^+^ cells due to lentiviral infection, which could be rescued by BCL-X_L_ overexpression (Figure 2F). The yield of GFP^+^ CD34^+^ cells was also mildly increased by BCL-X_L_ expression, albeit not significantly (Figure 2G). SR-1 addition did not affect the stem cell yield, neither in the presence nor in the absence of BCL-X_L_ overexpression (Figure 2G).

### 2.3. BCL-X_L_ Overexpression Increases the Reconstitution Potential of In Vitro Expanded Stem and Progenitor Cells

**Figure 3:**
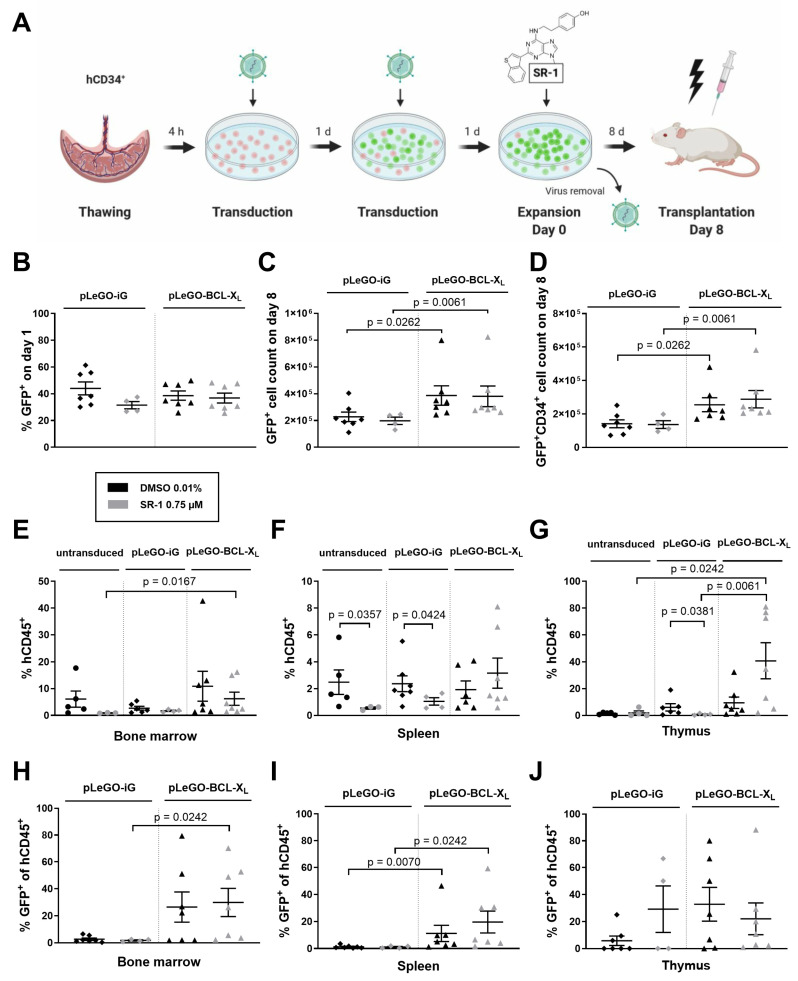
Cells expanded in the presence of BCL-X_L_ overexpression have a survival advantage in vivo. (**A**) Experimental design of xenotransplantation experiment. 10^5^ CD34^+^ cells were transduced twice with pLeGO-iG (control) or pLeGO-BCL-X_L_ lentivirus (MOI = 10). Subsequently, the cells were cultured in the presence of cytokines (100 ng/mL TPO, SCF, FLT3L, and IL-6) and treated with 0.75 µM SR-1 or DMSO 0.01% (control). The transduction efficiency was determined by flow cytometry on day 1. The progeny of 1 × 10^5^ CD34^+^ cells were counted and analyzed by flow cytometry on day 8 of culture prior to transplantation. (Figure 3B–D) (**B**) % GFP^+^ on day 1. (**C**) GFP^+^ cell count on day 8. (**D**) GFP^+^ CD34^+^ cell count on day 8. On day 8 of culture, 9 × 10^5^ expanded cells were transplanted retro-orbitally into sublethally irradiated Rag2^−/−^γc^−/−^ mice. Eight weeks after transplantation, recipient mice were sacrificed and single cell suspensions of their hematopoietic and lymphatic organs were analyzed by flow cytometry. (Figure 3E–J) (**E**) Total human engraftment in bone marrow. (**F**) Total human engraftment in spleen. (**G**) Total human engraftment in thymus. (**H**) % GFP^+^ cells of human CD45^+^ cells in bone marrow. (**I**) % GFP^+^ cells of human CD45^+^ cells in spleen. (**J**) % GFP^+^ cells of human CD45^+^ cells in thymus. Bars represent means of *n* = 4–7 from 8 independent experiments for (**B**–**D**), means of *n* = 3–7 from 8 independent experiments ± SEM for (**E**–**G**), and means of *n* = 4–7 from 8 independent experiments ± SEM for (**H**–**J**). Significant *p* values are indicated (Mann–Whitney Test).

To address the in vivo engraftment potential of human HSPCs after in vitro expansion in combination with apoptosis inhibition via BCL-X_L_ overexpression, Rag2^−/−^γc^−/−^ mice were xenotransplanted according to the scheme in Figure 3A. Based on the above data, we decided to expand cells for 8 days using cytokines and 0.75 µM SR-1 as described earlier. The transduction efficiency was comparable in both groups (32% and 44% with pLeGO-iG +/− SR-1 vs. 37% and 39% with pLeGO-BCL-X_L_ +/− SR-1; Figure 3B). While no effect of SR-1 on numbers of total GFP^+^ cells and GFP^+^ CD34^+^ cells was noted, a significant expansion of both cell populations could be obtained by BCL-X_L_ overexpression (e.g., 1.4 × 10^5^ GFP^+^ CD34^+^ cells with pLeGO-iG + DMSO vs. 2.6 × 10^5^ cells with pLeGO-BCL-X_L_ + DMSO, *p* = 0.0262; Figure 3C,D). For additional characteristics of the expanded cells see Supplementary Figure S2. On day 8 of expansion, 9 × 10^5^ cells from each group were transplanted into 5-week-old Rag2^−/−^γc^−/−^ mice after sublethal irradiation with 3 Gy. Eight weeks after transplantation, the recipient animals were sacrificed, single cell suspensions were obtained from their hematopoietic and lymphatic organs (i.e., bone marrow, spleen, thymus), and analyzed by flow cytometry. Total human engraftment in bone marrow, reflected by the percentage of human CD45^+^ cells, seemed to be reduced, although not significantly, when cells were pretreated with SR-1 (Figure 3E). No significant differences were observed between untransduced and transduced cells. However, the combination of SR-1 pretreatment and BCL-X_L_ overexpression was significantly superior to the SR-1 pretreatment alone (Figure 3E). Importantly, lentivirally transduced cells only engrafted when BCL-X_L_ was overexpressed (Figure 3H). Splenic engraftment was significantly reduced when cells were pretreated with SR-1 (Figure 3F) or transduced with control lentivirus (GFP^+^, Figure 3I). Surprisingly, the generally poor thymic engraftment was improved when SR-1 pretreated cells were overexpressing BCL-X_L_ (Figure 3G,J).

### 2.4. Inhibitors Targeting Caspases and Necroptosis Show no Beneficial Effects during Ex Vivo Expansion

**Figure 4:**
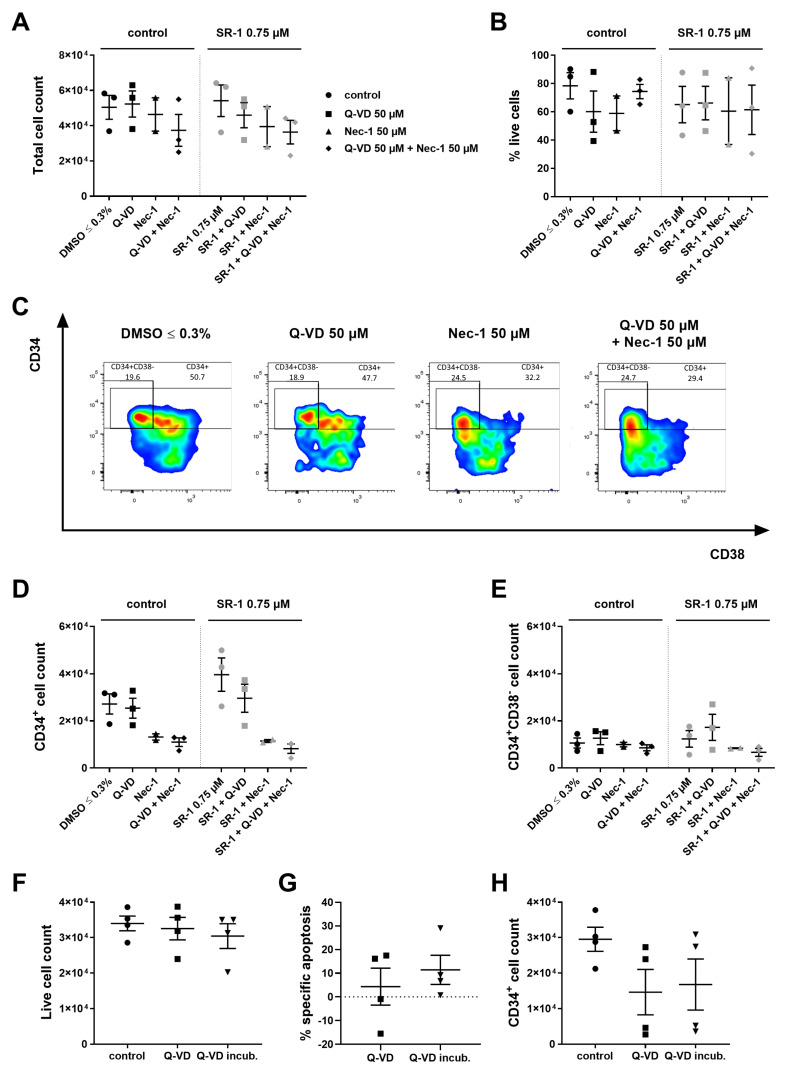
Caspase and necroptosis inhibition are not beneficial for ex vivo expansion. 10^4^ CD34^+^ cells were cultured in the presence of cytokines (100 ng/mL TPO, SCF, FLT3L, and IL-6) and treated with 50 µM Q-VD-OPh (Q-VD) or 50 µM Necrostatin-1 (Nec-1) and 0.75 µM SR-1 or DMSO ≤ 0.3%. Cells were counted and analyzed by flow cytometry on day 4 of culture. (Figure 4A–E) (**A**) Total cell count. (**B**) % live cells. (**C**) Representative FACS plots of CD34 and CD38 expression on day 4. (**D**) CD34^+^ cell count. (**E**) CD34^+^CD38^−^ cell count. 5 × 10^4^ freshly purified CD34^+^ cells were frozen according to the normal freezing protocol (control), treated with 50 μM Q-VD added directly to the freezing medium, or incubated for 30 min with 50 μM Q-VD before additionally being treated with 50 μM Q-VD added directly to the freezing medium (Q-VD incub.). After 5 to 12 months in liquid nitrogen at −196 °C, the cells were thawed according to the normal protocol, counted, and flow cytometric analysis was performed. (Figure 4F–H) (**F**) Live cell count. (**G**) Specific apoptosis compared to the normal freezing protocol. (**H**) CD34^+^ cell count. Bars represent means of *n* = 2–3 from 3 independent experiments ± SEM for (**A**−**E**) and means of *n* = 4 from 4 independent experiments ± SEM for (**F**–**H**). Significant *p* values are indicated (Mann–Whitney Test).

Our experiments show that lentiviral transduction severely limits survival and engraftment of human HSPCs. In addition, continuous overexpression of antiapoptotic BCL-2 proteins was shown to increase the risk of lymphoma and autoimmunity in transplanted individuals [8,9]. We thus decided to use techniques of transient apoptosis inhibition during ex vivo expansion and early engraftment. Earlier we showed that adenoviral and protein transduction of BCL-X_L_ acts transiently but is technically difficult in human CD34^+^ cells [7]. We now chose to inhibit apoptosis differently by using the pan-caspase inhibitor quinolyl-valyl-O-methylaspartyl-[-2,6-difluorophenoxy]-methyl ketone (Q-VD-OPh) known to inhibit caspase-dependent apoptosis. Since necroptosis is another form of cell death upon caspase inhibition, we included the RIPK1 inhibitor necrostatin 1 (Nec-1) in our studies [10,11]. We have shown earlier that treatment with these compounds protected murine HSCs from apoptosis induced by cytokine deprivation in vitro [7]. For HSPC expansion, 50 µM Q-VD-OPh, 50 µM Nec-1, or the combination of both were used. Analysis on day 4 of culture unexpectedly revealed no increase in the total cell count or the cell viability (Figure 4A,B), but the treatment changed the immune phenotype of the cells (Figure 4C). Similarly, no beneficial effects on CD34^+^ and the most immature CD34^+^CD38^−^ cells were obtained by Q-VD-OPh or Nec-1 treatment (Figure 4D,E; *n* = 2–3, no statistics performed).

In another approach, we assessed the effect of caspase inhibition during freezing and thawing of human HSPCs with the aim to increase stem cell yield in the case of HSCT with cryoconserved donor cells. Freshly isolated CD34^+^ cells were incubated with 50 µM Q-VD-OPh for 30 min before freezing, or were directly frozen in cryopreservation medium supplemented with the compound according to our normal freezing protocol. Analysis after thawing revealed that caspase inhibition did not influence the survival of CD34^+^ cells (Figure 4F–H). Also regarding the freezing procedure, caspase inhibition did not prove suitable to achieve higher viability and hence higher cell counts.

## 3. Discussion

Improving hematopoietic reconstitution after HSCT is the aim of numerous different strategies. These include approaches to improve HSPC mobilization and collection, ex vivo expansion, homing to the recipient’s stem cell niche as well as methods to accelerate engraftment, for example via double-unit cord blood transplantation or co-infusion with accessory cells [12]. Presumably, the combination of different approaches is most promising in order to maximize the available donor cell numbers. We decided to combine two methods: ex vivo stem cell expansion and cell death inhibition.

Ex vivo cultured, proliferating HSCs are especially susceptible to apoptosis [13]. One possible explanation is that errors in DNA replication occur during each cell cycle. If the genomic damage is beyond repair, cell death mechanisms are triggered, a fact which is particularly important for strongly proliferating cells [14]. For expanded human CD34^+^ cells, a correlation between apoptosis and the number of cell divisions has been reported [15]. Furthermore, earlier transplantation experiments have shown that more apoptosis occurred in recipient animals after transplantation of expanded HSCs compared to quiescent donor cells [16]. Another reason for increased susceptibility to apoptosis is the lack of physiological prosurvival signals under in vitro conditions, which are normally provided by the stem cell niche, such as humoral factors, cell-cell, or cell-matrix contacts [17]. We now aimed at inhibiting apoptosis during both ex vivo expansion and HSCT based on our previous experience with apoptosis inhibition. Briefly, we have demonstrated that a significant amount of HSPCs are lost during HSCT due to apoptotic cell death mediated via the BH3-only proteins BIM and BMF [6]. Furthermore, we have proven that even short-term apoptosis inhibition during 7–10 days after transplantation is sufficient to improve donor cell performance and thus engraftment [7]. This approach conferred a survival advantage during the most critical phase of HSCT and at the same time minimized the risk of neoplastic transformation [18].

Our combined approach reveals that apoptosis resistance via overexpression of BCL-X_L_ is effective during HSPC expansion to increase both stem cell fraction and engraftment. However, substantial limitations were noted. Despite various attempts to optimize our culture conditions based on the protocols of Boitano et al. [3], we were not able to reproduce earlier findings, and expansion via the small molecule SR-1 proved underwhelming. Meanwhile, the corresponding phase II trial with this compound has been withdrawn due to IRB disapproval (NCT02765997) [19]. In the xenotransplantation experiments, we observed an exceptionally high thymic engraftment of several samples treated with pLeGO-BCL-X_L_ and SR-1. We hypothesize that this combination might have driven differentiation towards the lymphoid lineage. Interestingly, aryl hydrocarbon receptor antagonism has earlier been reported to enhance lymphoid cell differentiation from human embryonic stem cells [20]. Such a differentiation bias needs to be avoided for a safe therapeutic application of ex vivo expansion in the clinical setting.

With respect to apoptosis, severe toxicity of lentiviral transduction, observed both in vitro and in vivo, hampered our approach. Lentiviral toxicity could be partially compensated by BCL-X_L_ overexpression indicating that both intrinsic apoptosis and other forms of cell death contribute to HSPC loss after lentiviral infection. Additionally, the transduction efficiency was variable and sometimes very low. As a consequence, we could not demonstrate in all experiments a significant ex vivo HSPC expansion based on absolute cell counts. Currently, lentiviruses are one of the key vector platforms used for gene therapy [21]. Continuous improvements in lentiviral vectors might substantially reduce toxic side effects such as induction of cell death. Still, the use of integrating viruses resulting in continuous apoptosis inhibition cannot be considered safe in this setting due to the risk of long-term sequelae such as autoimmunity and leukemia [8,9].

Irrespective of the technical limitations associated with SR-1 on the one hand and the lentiviral transduction on the other hand, we can conclude from our studies that apoptosis inhibition will be feasible and reasonable during stem cell expansion induced by other compounds. A promising candidate for HSPC expansion might be the pyrimidoindole derivative UM171, which is currently being evaluated in several clinical trials after favorable results in a phase 1–2 safety and feasibility study [22,23]. Alternatives for our lentiviral transduction approach could be BCL-X_L_ mRNA used in analogy to the recently established mRNA vaccines or direct BAX/BAK inhibitors, which are currently being developed [24]. Cell death inhibition on the level of caspases or the necroptosis machinery, in contrast, proved not suitable in this study. One explanation might be that non-lethal caspase signaling exerts physiological roles in the cell, for example during proliferation and differentiation [25]. Considering the complex cellular functions of caspases and the interconnectivity between cell death and other signaling pathways, the therapeutic application of caspase inhibitors for various diseases remains challenging [26]. Importantly, for all newly tested combination therapies, functional assays (such as colony-forming unit assay or aldehyde dehydrogenase detection assay) and xenograft transplantations will be required prior to therapeutic use in order to demonstrate that the pre-transplantation treatment does not interfere with the stem cell potential to self-renew and differentiate into all hematopoietic lineages.

In conclusion, apoptosis inhibition by transient overexpression of BCL-X_L_ or other means is a promising approach for ex vivo expansion of HSPCs and, possibly, their genetic modification for gene therapy applications.

## 4. Materials and Methods

### 4.1. Cell Isolation and Culture

Human umbilical cord blood was collected from the placental vein after Caesarean section with informed consent of the parents. Approval had been obtained from the local ethics committee. Human CD34^+^ cells were enriched to a purity of over 90% using microbead-based magnetic separation (CD34 MicroBead Kit; Miltenyi Biotech, Bergisch Gladbach, Germany). Purified cells were directly used or slowly frozen (1 °C/min) in 500–1000 µL CryoStor^®^ CS10 medium (Sigma-Aldrich, St. Louis, MO, USA) to −80 °C using freezing containers with isopropyl alcohol. For long-term storage, the vials were transferred to liquid nitrogen (−196 °C) after at least 3 days. Fresh or thawed CD34^+^ cells were cultured in serum-free medium (StemPro^®^-34 with 1× StemPro^®^-34 Nutrient Supplement or StemSpan) supplemented with the recombinant human cytokines FLT3L, IL-6, SCF, and TPO (100 ng/mL each; ImmunoTools, Friesoythe, Germany), and StemRegenin 1 at the indicated concentrations (STEMCELL Technologies, Vancouver, BC, Canada) or DMSO 0.01% or ≤0.3% (Sigma-Aldrich). Cell density was kept between 10^5^–10^6^ cells. The caspase inhibitor Q-VD-OPh (MP Biomedicals, Illkirch-Graffenstaden, France) and the necroptosis inhibitor Nec-1 (Merck, Darmstadt, Germany) were used at a concentration of 50 µM each.

### 4.2. Lentiviral Transduction

BCL-X_L_ was overexpressed in the lentiviral gene ontology (LeGO) vector pLeGO-iG under control of the spleen focus-forming virus (SFFV) promoter kindly provided by Heike Pahl, University Medical Center Freiburg [27]. For the generation of lentiviruses, lentiviral backbone plasmids were transfected into HEK 293T cells (kindly provided by Heike Pahl, University Medical Center Freiburg) with the ProFection^®^ Mammalian Transfection System Kit (Promega, Madison, WI, USA) using the CaPO_4_ method. BCL-X_L_ expression was confirmed in HeLa cells (kindly provided by Heike Pahl, University Medical Center Freiburg; Supplementary Figure S1). Human CD34^+^ cells were incubated in stem cell medium supplemented with the previously mentioned cytokines for 4 h, and subsequently incubated with lentiviruses for 48 h (2 × 24 h, MOI = 10). After transduction, the cells were resuspended in fresh stem cell medium supplemented with cytokines and used for further experiments. The transduction efficacy was determined by flow cytometric analyses of GFP expression. Lentiviral work was performed according to S2 safety regulations.

### 4.3. Xenotransplantation

All experiments were performed in accordance with the guidelines of German laws regarding animal experiments and approved by the local committee (RP Freiburg/Germany). For xenotransplantation experiments, 10^5^ human CD34^+^ cells per condition were lentivirally transduced and expanded for 8 days before transplantation. Five-week-old Rag2^−/−^γc^−/−^ mice were irradiated sublethally (3 Gy), and 6–8 h afterwards 9 × 10^5^ CD34^+^ cells were transplanted intravenously into the retrobulbar venous plexus [28]. Eight weeks after transplantation, the mice were sacrificed and the hematopoietic and lymphatic organs were analyzed by flow cytometry.

### 4.4. Flow Cytometric Analysis

Single cell suspensions of expanded CD34^+^ cells or of hematopoietic or lymphatic organs were surface-stained with monoclonal antibodies conjugated with the indicated fluorochromes or with biotin. The antibodies for human cell-surface markers were: hCD34 PE (AC136), Miltenyi Biotec; hCD34 PE-Cy7 (581), BioLegend, San Diego, CA, USA; hCD38 APC (HIT2 and IB6), BioLegend and Miltenyi Biotec; and hCD45 biotin (HI30), BD Biosciences, Franklin Lakes, NJ, USA. Biotinylated antibodies were detected using streptavidin coupled to APC-Cy7 (BioLegend). To distinguish murine cells, mCD45 PE-Cy7 (30-F11, eBioscience, San Diego, CA, USA) was used. Apoptosis was determined by staining with 7-AAD (Sigma-Aldrich) and Annexin-V (FITC, BD Biosciences; Alexa Fluor^®^ 647, BioLegend). To determine specific apoptosis, the values were normalized for basal apoptosis according to the following equation: (apoptosis of treated cells—apoptosis of control)/(100—apoptosis of control). Flow cytometric analyses were performed on BD LSRFortessa.

### 4.5. Statistical Analysis

Statistical analysis was performed with the non-parametric Mann–Whitney test (unpaired) in GraphPad Prism 10 (GraphPad Software, Boston, MA, USA). *p*-values ≤ 0.05 were considered statistically significant.

## Data Availability

Dataset available on request from the authors.

## Supplementary Materials

The following supporting information can be downloaded at: https://www.mdpi.com/article/10.3390/ijms25074105/s1.
