# Supplementary material for: Lentivirus-Mediated BCL-XL Overexpression Inhibits Stem Cell Apoptosis during Ex Vivo Expansion and Provides Competitive Advantage Following Xenotransplantation
Source: Int J Mol Sci. 2024 Apr 7;25(7):4105. doi: 10.3390/ijms25074105 (PMC11012376; doi:10.3390/ijms25074105)
Supplement: Supplementary file 1 [file ijms-25-04105-s001.zip › ijms-2869651-supplementary.pdf]

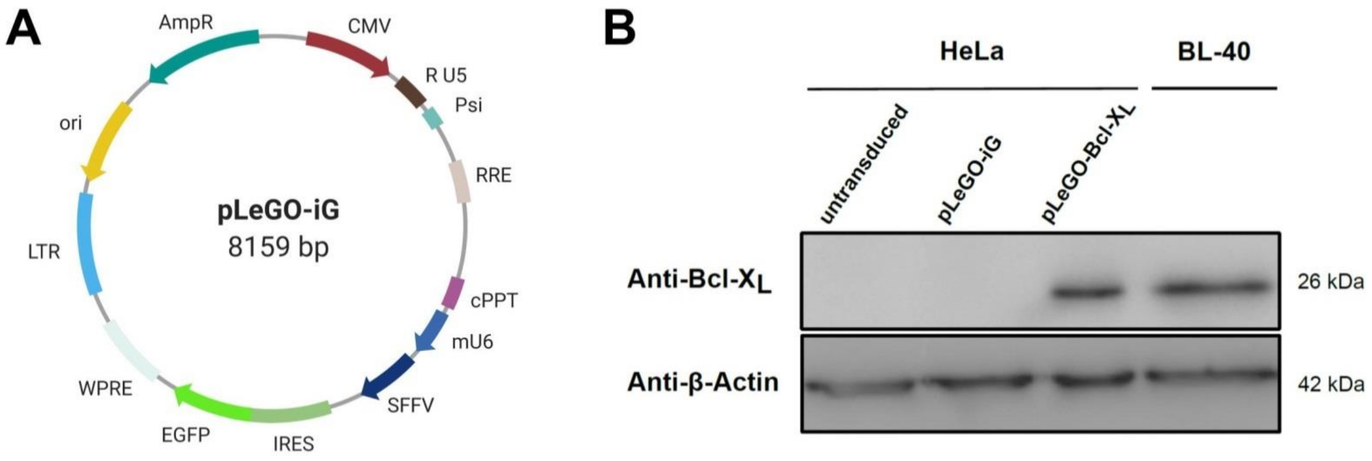

Supplementary Figure 1: Lentiviral transduction with pLeGO-iG and pLeGO-BCL-X_L.

A: Vector map of pLeGO-iG. B: $1x10^6$ HeLa cells were lentivirally transduced with pLeGO-iG (control) or pLeGO-BCL-X_L (MOI = 10 - 20), or remained untransduced. After three days the cells were pelleted, proteins were isolated, and the BCL-X_L protein content was analyzed by western blot. The Burkitt lymphoma cell line BL40 served as a positive control, β-actin as a loading control.

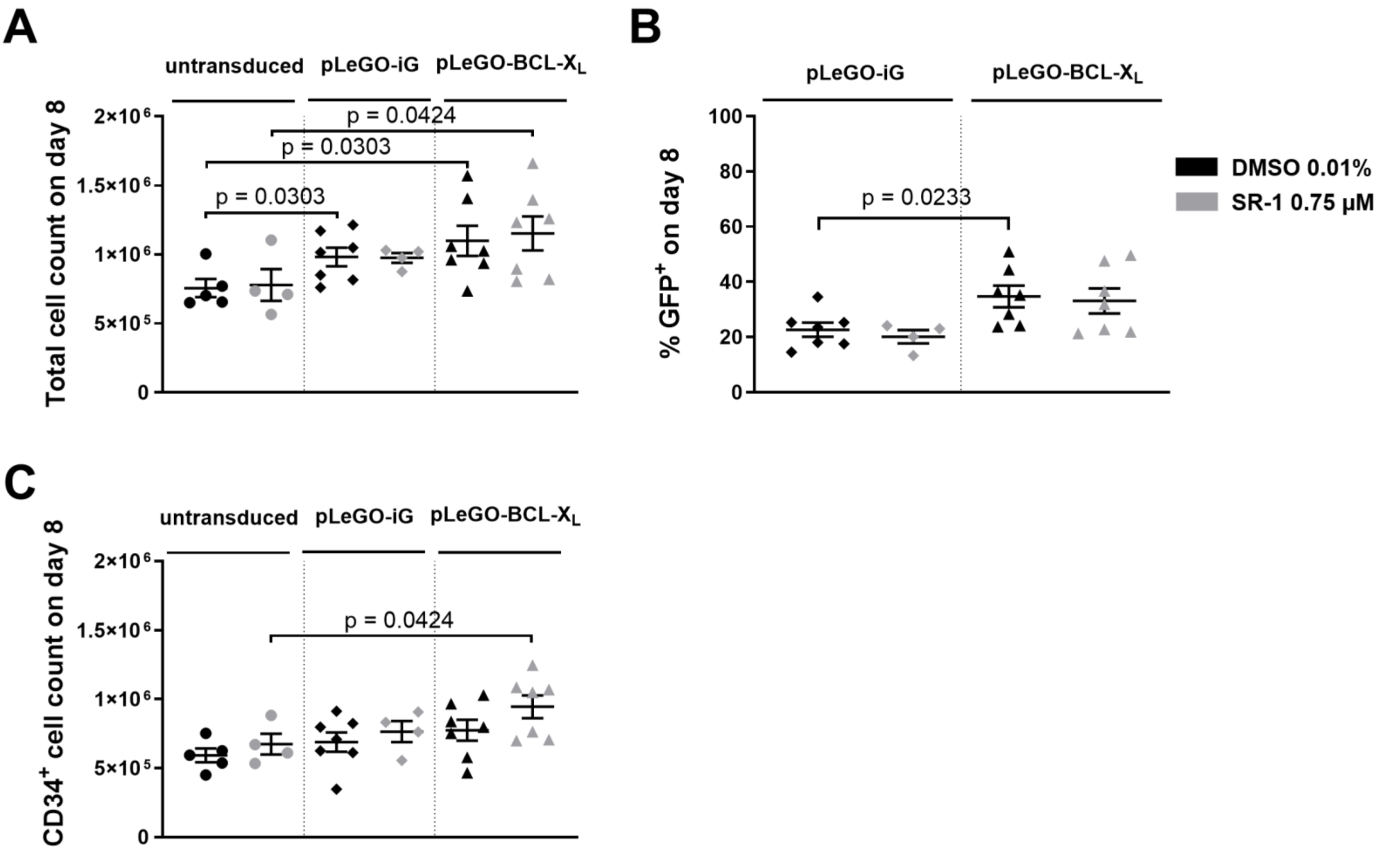

Supplementary Figure 2: Characteristics of lentivirally transduced and expanded CD34$^+$ cells prior to transplantation.

For experimental design see Figure 3A. The progeny of 1×10^5 CD34$^+$ cells was counted and analyzed by flow cytometry on day 8 of culture prior to transplantation. A: Total cell count on day 8. B: % GFP$^+$ of live cells on day 8. C: CD34$^+$ cell count on day 8. Bars represent means of n = 4 - 7 from 8 independent experiments ±SEM. Significant p values are indicated (Mann-Whitney Test).
